# Dieulafoy's Lesion in a Duodenal Diverticulum: A Case Report and Literature Review

**DOI:** 10.7759/cureus.46584

**Published:** 2023-10-06

**Authors:** Zolfekar A Yahia, Saud M Almutawa, Bassam N Bantan, Muhannad S Alghamdi

**Affiliations:** 1 Department of Gastroenterology, Alnoor Specialist Hospital, Makkah, SAU; 2 College of Medicine, Umm Al-Qura University, Makkah, SAU

**Keywords:** duodenal diverticular dieulafoy's lesion, literature review, case report, endoscopic management, hemostatic clip, hemorrhagic shock, acute gastrointestinal bleeding, duodenal diverticulum, dieulafoy's lesion

## Abstract

This report presents a case of Dieulafoy's lesion (DL), a rare and serious gastrointestinal condition, which occurred unusually in a duodenal diverticulum and highlights the diagnostic and management complexities associated with it. A literature review of six similar cases revealed commonalities in presentation, diagnosis, and management, emphasizing the complexities in identifying and handling this rare manifestation of DL. The findings emphasize the need for clinical vigilance and further research into optimizing strategies for diagnosing and managing this rare condition.

## Introduction

Dieulafoy’s lesion (DL), also referred to as a cirsoid aneurysm or submucosal arterial malformation, is a rare yet severe etiology of acute gastrointestinal (GI) bleeding. This condition presents a significant clinical challenge due to its association with high morbidity and mortality rates [1]. The condition was first identified by Gallard in 1884, adding to our understanding of uncommon causes of GI bleeding [2].

The exact incidence of Dieulafoy's lesion in Saudi Arabia remains undetermined due to data scarcity. However, on a global scale, DL accounts for approximately 5% of acute GI bleeding cases, underlining its clinical importance despite its rarity. The condition can manifest across all age groups but is more frequently observed in elderly individuals, particularly those with preexisting comorbidities such as hypertension, diabetes mellitus, and chronic kidney disease. Additionally, a slight male predominance has been noted in the occurrence of DL [1,3].

Endoscopy is considered the gold standard in treating DL, boasting a success rate exceeding 90%. This technique allows for direct visualization and intervention, markedly reducing the risk of persistent or recurrent bleeding. For cases unresponsive to endoscopic treatment, an angiographic approach can be employed to embolize the bleeding vessel, offering an alternative route for managing this complex condition [4]. The following case report delves into the management of a challenging case of DL within a duodenal diverticulum, providing valuable insights into the diagnosis and treatment of this rare condition.

## Case presentation

A 63-year-old male patient from Indonesia was urgently admitted to the emergency room (ER) exhibiting symptoms of hematemesis and melena. His medical history included dyspepsia. The patient, who had traveled to Makkah, Saudi Arabia, for pilgrimage, was unaccompanied by any family members or friends.

Upon evaluation, the patient appeared disoriented and exhibited pallor. His vital signs indicated a heightened state of distress, with a heart rate of 127 bpm, blood pressure at a low 88/54 mmHg, a body temperature of 36.8°C (98.24F), and an O_2_ saturation of only 90%. Additionally, the patient presented with edema in his limbs, although the remainder of his physical examination was unremarkable. Laboratory investigations were promptly initiated upon admission, the results of which are detailed in Table 1.

**Table 1:** Laboratory investigations BUN: blood urea nitrogen; SGPT: serum glutamic pyruvic transaminase; ALT: alanine aminotransferase; SGOT: serum glutamic-oxaloacetic transaminase; AST: aspartate aminotransferase; CK: creatine kinase; CPK: creatine phosphokinase; LDH: lactate dehydrogenase

Test name	Result		Reference range
BUN	21.000 (H)		3.2 - 8.2 mmol/L
Creatinine	204.000 (H)		62 - 115 umol/L
Sodium (Na)	146.000 (H)		135 - 145 mmol/L
Albumin	19.000 (L)		34- 50 g/L
Bilirubin Total	4.000		0- 18.7 umol/L
Bilirubin Direct	2.000		0- 5 umol/L
SGPT/ALT	259.000 (H)		10 - 49 U/L
SGOT/AST	112.000 (H)		10 - 34 U/L
Amylase	204.000 (H)		25- 110 U/L
Potassium (K)	5.600 (H)		3.5 - 5.1 mmol/L
CK/CPK	36.000 (L)		45 - 170 U/L
LDH	193.000		80-230 U/L
Serum lipase	110. 000 (H)		13-60 U/L
Troponin-I	0.070 (H)		0-0.05 ug/L

Initial ER management included the administration of omeprazole for gastric acid suppression, paracetamol for symptomatic relief, and a transfusion of one unit of packed red blood cells (PRCB) to address the patient's anemia. His hyperkalemia and acute kidney injury (AKI) were managed with intravenous fluids and anti-hyperkalemic measures.

**Figure 1:**
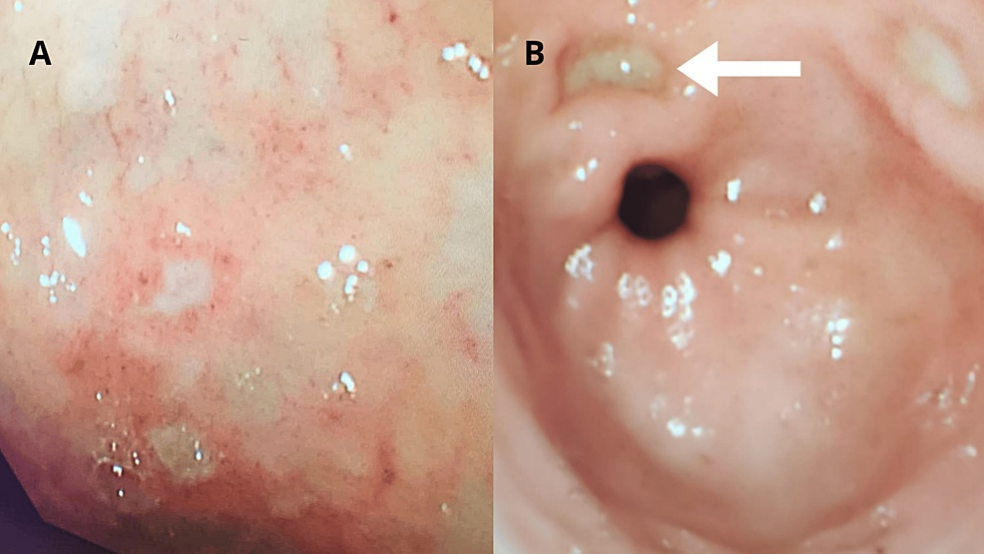
First endoscopy A: Endoscopic view of multiple clean-based ulcers located in the pre-pyloric area. B: Picture of the small deep ulcer inside the pyloric opening.

Endoscopic examination revealed a normal esophagus and an absence of blood in the stomach (Figure 1). However, the patient had multiple clean-based ulcers in the pre-pyloric area (Figure 1A). In the duodenum, multiple clean-based erosions were detected, as well as a small, deep ulcer with a black spot (Forrest class IIC) situated just inside the pyloric opening at the 2 o’clock position (Figure 1B). No active bleeding was observed during the procedure.

**Figure 2:**
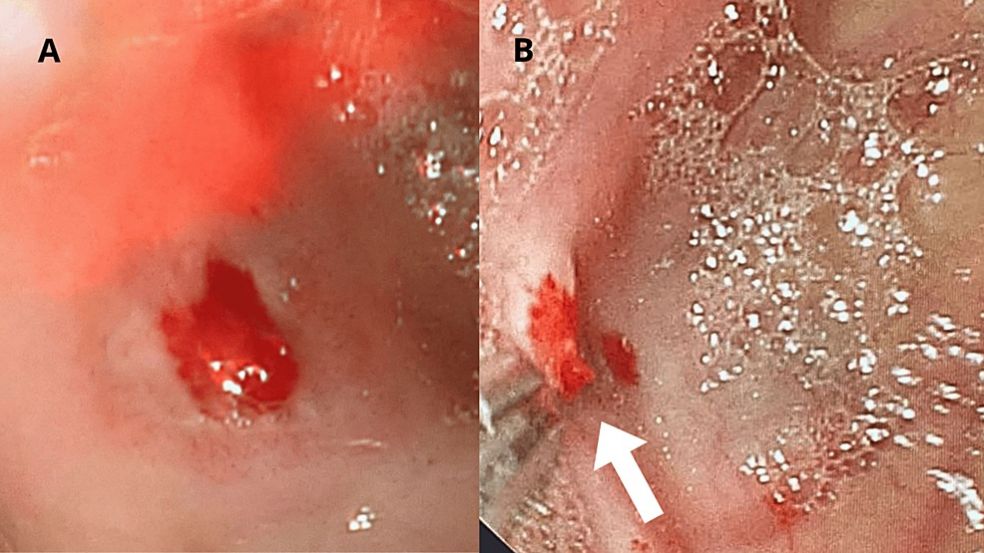
Second endoscopy A: Endoscopic view of bleeding Dieulafoy’s lesion inside the diverticulum prior to the application of hemostatic clips. B: Endoscopic view of the diverticulum post-application of hemostatic clips, showing successful control of the bleeding.

Despite initial stabilization and transfer to the general ward, the patient experienced re-bleeding and was subsequently moved to the Intensive Care Unit (ICU) due to hemorrhagic shock. A second endoscopy (Figure 2) revealed Dieulafoy’s lesion at the edge of a diverticulum with fresh blood emanating from the distal horizontal aspect of the duodenum (D3) (Figure 2A). Hemostasis was achieved with the application of a hemostatic clip (Figure 2B).

Whilst in the ICU, the patient was managed with fentanyl for pain, vasopressin for blood pressure control, and multiple units of PRCB. On the third day of ICU admission, he developed cellulitis, evident by erythema and swelling in both forearms, which was treated with ceftriaxone and daily dressing.

Upon significant improvement by the eighth day, the patient was transferred to the general ward, and from gastroenterology to the care of general medicine. Unfortunately, he developed a fever and was started on a course of vancomycin, meropenem, and amikacin. After the culture results showed candida in urine and klebsiella in blood, the course was changed to tigecycline and colistin after an infectious diseases consultation.

This case highlights the diagnostic challenges and management complexities associated with rare causes of UGIB, such as Dieulafoy's lesion.

## Discussion

Dieulafoy's lesion is a submucosal vessel that is dilated and aberrant and erodes the overlying epithelium. The submucosal artery is not able to branch properly within the stomach wall. Therefore, the caliber of the artery is in the range of 1 to 3 mm, about 10 times the usual caliber of the mucous capillaries [2].

While 90% of Dieulafoy's lesion cases are found in the proximal stomach, they may, albeit rarely, occur in other regions such as the duodenum, esophagus, and colorectal area [1,5]. The exact pathophysiology leading to the rupture and bleeding of these lesions remains uncertain, but one theory suggests a significant role of arterial thrombosis and subsequent necrosis [4,6].

In general, UGIB can be caused by various conditions, for example, esophagitis, peptic ulcers (gastric and duodenal), and gastric varices, in this case, the diagnosis of Dieulafoy's lesion was established by endoscopy.

Endoscopy has emerged as the preferred diagnostic modality, achieving success in identifying Dieulafoy's lesions in approximately 70% of patients [4]. In terms of treatment, it is possible to categorize endoscopic hemostatic procedures into three groups: (1) Thermal electrocoagulation, heat probe coagulation, and argon plasma coagulation; (2) Epinephrine injection and sclerotherapy; (3) Mechanical banding and hemoclips. With varying success rates, each technique has its advantages and disadvantages. Compared to the injection or thermal treatment method, endoscopic mechanical hemostatic methods are more effective and successful in achieving hemostasis. Also, combined endoscopic therapies have been reported to have a lower rate of re-bleeding than endoscopic monotherapy. The use of angiography for embolizing actively bleeding lesions is an option if endoscopic methods do not work. Embolization, however, carries the risk of ischemia. Surgical resection - once the first-line approach - has largely been replaced by endoscopic procedures due to advancements in the field. Today, surgical interventions, such as gastrotomy or gastrectomy, are reserved for a small subset of cases that prove unresponsive to endoscopic or angiographic methods [4].

We conducted a literature search using the PubMed database, specifically focusing on instances of Dieulafoy’s lesion occurring within a duodenal diverticulum. For our search, we used a combination of terms, including 'Dieulafoy’s lesion' and 'duodenal diverticulum'. From this process, we identified a total of seven relevant cases. However, one case was excluded due to limited accessibility, resulting in a final sample of six cases [7-12].

In our review, we discovered that DL inside a duodenal lesion typically involved patients older than 67 years, with an average age of 79 years (Table 2). This condition was reported to affect males in 100% of the cases (Table 2). The most common presentations were melena and hematemesis; one of the reported cases also presented with epigastric pain [7]. In our review, we noticed that all but one patient had a co-morbid condition [8], with hypertension being the most common (33%). A range of comorbidities, including diabetes mellitus, ischemic heart disease, liver cirrhosis, chronic kidney disease, heart failure, and atrial fibrillation, were reported across the sample. (Table 2).

**Table 2:** Clinical features

Clinical Feature	N (%)
Gender	
Male	6 (100)
Age	
65 - 69	1 (16)
70 - 75	2 (33)
76 - 85	1 (16)
86 - 99	2 (33)
Comorbid Condition	
Hypertension	2 (33)
Diabetes Mellitus	1 (16)
Chronic Kidney Disease	1 (16)
Liver Cirrhosis	1 (16)
Ischemic Heart Disease	1 (16)
Atrial Fibrillation	1 (16)
Heart Failure	1 (16)
Mitral Regurgitation	1 (16)
Dyslipidemia	1 (16)

All the patients underwent an initial endoscopy, which successfully revealed the lesions in four out of the six patients. One patient required a second endoscopy [9] while another required an abdominal CT scan [10].

The patients were managed differently mostly by hemostatic clips (83%) and adrenaline injections (50%), one patient had argon plasma electrocoagulation alongside the adrenaline injection [10], and another patient was managed by epinephrine injection, fibrin glue, and N-butyl-2-cyanoacrylate/lipiodol moisture (Table 3) [9].

**Table 3:** Management methods

Therapeutic modality	N (%)
Hemostatic Clips	5 (83)
Adrenaline Injection	3 (50)
Argon Plasma Electrocoagulation	1 (16)
Fibrin Glue	1 (16)
N-butyl-2-cyanoacrylate / Lipiodol Moisture	1 (16)

While our case report provides valuable insights into the diagnosis and management of Dieulafoy's lesion in a duodenal diverticulum, we acknowledge several limitations, largely stemming from circumstances specific to the patient and context.

Communication challenges

A significant challenge was the existing communication barrier. The patient was in a state of compromised consciousness, marked by delirium and confusion. This condition severely curtailed our ability to gather vital information regarding his symptoms. This issue was further compounded by the language barrier, which could have potentially resulted in misinterpretations or omissions of critical information.

Incomplete medical history

Another major limitation was the inability to access the patient's comprehensive past medical and surgical history. The patient traveled to Saudi Arabia for a pilgrimage without any accompanying family members. This situation made it challenging to gather detailed information regarding his previous health conditions or surgical interventions, which could have potentially influenced our diagnostic and treatment decisions.

Furthermore, there was a black spot on the endoscopic image of the deep duodenal ulcer, but a clear picture could not be obtained. 

## Conclusions

This case highlights the challenges of diagnosing and managing a patient presenting with acute gastrointestinal bleeding due to a Dieulafoy's lesion. The lesion was successfully identified and treated endoscopically, emphasizing the utility of endoscopic interventions. The case also presented challenges with acute kidney injury and hyperkalemia, secondary to the bleeding, which were adeptly managed. The patient's ongoing inpatient treatment for an infection and engagement in physiotherapy sessions underline the importance of a comprehensive and holistic approach to patient care.
